# A pilot study of a targeted cognitive intervention for cancer survivors

**DOI:** 10.1007/s00520-025-09321-z

**Published:** 2025-03-07

**Authors:** Joaquin A. Anguera, Karin Snowberg, Steven M. Paul, Bruce A. Cooper, Kate Oppegaard, Carolyn Harris, Christine Miaskowski

**Affiliations:** 1 School of Medicine, University of California, San Francisco, CA USA; 2 School of Nursing, University of California, San Francisco, CA USA; 3 VA Portland Health Care System, Portland, OR USA; 4 School of Nursing, University of Pittsburgh, Pittsburgh, PA USA; 5 Department of Physiological Nursing, University of California, San Francisco, 490 Illinois Street, Floor 12, San Francisco, CA 94143 USA

**Keywords:** Attention, Cancer, Cognitive impairment, Cognitive intervention, Multi-tasking, Working memory

## Abstract

**Purpose:**

The primary aims of this four week pilot randomized clinical trial (RCT) involving a targeted cognitive intervention (TCI, *n* = 25) compared to an expectancy matched active control intervention (EMACI, *n* = 24), in a sample of cancer survivors were to: determine recruitment and retention rates; evaluate preliminary efficacy to improve three objective measures of cognitive function (i.e., attention, working memory, multi-tasking); evaluate adherence rates for and satisfaction with the interventions, and evaluate for treatment-related adverse events (e.g., nausea, motion sickness).

**Methods:**

Cancer survivors were recruited from previous studies through email. Following a screening call, survivors who consented to participate were oriented to the study measures and procedures via Zoom. Survivors were randomized to the TCI or EMACI and mailed an iPad with the software for their specific intervention and the Adaptive Cognitive Evaluation Explorer (ACE-X, the objective measure of cognitive function). Survivors used the intervention for 25 min per day at least 5 days per week. Differences in objective measures of attention, working memory, and multi-tasking were evaluated using multilevel regression analyses.

**Results:**

For the sustained attention measure, a significant cross-level interaction was found in favor of the TCI group. While improvements in multi-tasking occurred in both groups, while not statistically significant, the trend was larger for the TCI group. Equally important, in both groups, adherence with the intervention was high and adverse effects were minimal.

**Conclusions:**

These preliminary findings provide promising evidence of feasibility, acceptability, and efficacy that warrant evaluation in a RCT with a larger sample of cancer survivors.

## Introduction

As noted in a paper that called for a “Neuroscience Approach to Cancer Related Cognitive Impairment (CRCI)” [1], Dr. Horowitz from the National Cancer Institute noted that CRCI “is a term used to describe the constellation of cognitive difficulties experienced by cancer survivors”. Of note, approximately 75% of cancer survivors report CRCI following cancer treatment [2–4]. CRCI has a negative impact on survivors’ ability to: work, carry out routine activities, and engage in social and family relationships [5].

The cognitive domains associated with CRCI are assessed using a variety of self-report and objective measures [6, 7]. The most common self-reported problems in survivors include: distraction, forgetfulness, and difficulties with attention and multi-tasking [4]. In terms of objective, performance-based measures, the specific domains affected include: attention, working memory, processing speed, and learning and memory [8, 9].

While CRCI is a significant problem for survivors, treatment recommendations in the National Comprehensive Cancer Network’s Survivorship Guideline are limited to: education and counseling; self-management strategies; medications (e.g., psychostimulants); routine physical activity; psychotherapy; and/or cognitive rehabilitation [10]. In terms of self-management strategies and cognitive rehabilitation, a recent network analysis of randomized clinical trials summarized the effects of six neuropsychological interventions on subjective and objective measures of cognitive function in adult patients with non-central nervous system cancers [11]. The neuropsychological interventions included: cognitive training, cognitive rehabilitation, cognitive behavioral therapies (CBT), meditation/mindfulness-based interventions, psychoeducation, and supportive care. In terms of objective measures of CRCI, this network meta-analysis found that cognitive training was the most effective intervention to manage deficits in verbal memory, verbal fluency, and processing speed; meditation/mindfulness-based interventions were the most effective for enhancing attention; and psychoeducation interventions were the most effective for managing deficits in executive function. In terms of subjective measures of CRCI, CBT was the most effective intervention. However, the authors noted that because most of the studies were done with patients with breast cancer; some of the trials had a high risk of bias; and a wide range of subjective and objective measures of CRCI were used across the trials, additional research is warranted to be able to draw definitive conclusions.

Evidence to support the efficacy of pharmacological interventions for CRCI is extremely limited [12, 13] In addition, many of these drugs have deleterious side effects (e.g., dizziness) that can impair survivors’ ability to function. In terms of physical activity and exercise, across several reviews [14–17], the majority of studies reported modest improvements in CRCI following an exercise intervention. However, several limitations were noted, including: inconsistent measures of CRCI and variability in the types and duration of the exercise interventions.

An extremely promising approach to enhance cognitive function, that is based on the most current findings in cognitive neuroscience, is the use of custom-designed video games [18]. This type of targeted cognitive intervention (TCI) can harness the plasticity of the brain (i.e., the brain’s ability to modify its function, structure, and chemistry in response to new experiences) [18]. In a recent systematic review of sixteen studies that evaluated computer-based cognitive training interventions [19], most of the programs focused on multiple domains of cognition and were home-based. The length of the programs ranged from three to 24 weeks, with a frequency of one to five sessions per week that lasted from 20 min to two hours. Eleven of the sixteen studies reported improvements in objective measures of cognitive function (e.g., memory, attention, processing speed). The authors concluded that while this type of intervention holds promise, definitive conclusions cannot be drawn because: sample sizes were relatively small; components and doses of the various computer-based interventions were not comparable; assessors were not blinded to the intervention; and none had an expectancy matched active control intervention (EMACI) group. However, the overall findings suggest that additional research is warranted to evaluate the efficacy of computer-based cognitive training interventions for CRCI in survivors.

Our pilot study was designed to evaluate the preliminary efficacy of a TCI that demonstrated efficacy in enhancing cognitive function in older adults [20]. The primary aims of this four week pilot randomized clinical trial (RCT) involving a TCI (i.e., AKL-T01, *n* = 25) compared to an EMACI (i.e., AKL-T09, *n* = 24), in a sample of cancer survivors were to: determine recruitment and retention rates; evaluate preliminary efficacy to improve three objective measures of cognitive function (i.e., attention, working memory, multi-tasking); evaluate adherence rates for and satisfaction with the interventions, and evaluate for treatment-related adverse events (e.g., nausea, motion sickness).

## Methods

### Sample recruitment, screening, and enrollment procedures

Invitation emails were sent to cancer survivors who participated in our previous descriptive studies [21–23]; gave permission to be contacted for future studies; and had an Attentional Functional Index (AFI) [24] score of < 7.5 in a previous study. These emails contained a link to a pre-screen survey that collected contact and eligibility information. Survivors were included if they were able to read, write, and understand English; had an internet or WiFi connection; had access to a telephone or Zoom for the screening interview and educational session about how to do the cognitive assessment and use the application; and had an AFI score of < 7.5 on the enrollment assessment. Previous research determined the following categorization for AFI scores: < 5.0 = low levels of cognitive function, 5.0 to 7.5 = moderate levels of cognitive function, and > 7.5 = high levels of cognitive function [25]. Survivors were excluded if they were: receiving active treatment for cancer recurrence; had significant cognitive impairment as indicated by their AFI score (< 3.0); and/or had sensory or motor deficits that prevented them from doing the assessment and using the TCI application.

Study details were explained during a screening call. If the survivor indicated his/her willingness to participate, an orientation Zoom meeting was scheduled. After the screening call was completed, the survivor was emailed a survey link that contained a digital consent form and the study’s self-report measures. Following completion of the enrollment measures, survivors were randomized to the TCI or EMACI by the study statistician. Survivors were mailed an iPad with the software for their specific intervention and the Adaptive Cognitive Evaluation Explorer (ACE-X) instrument installed [26]. Survivors were not given log in credentials in advance. Therefore, they were not able to log into the applications until the orientation meeting.

### Instruments

Survivors completed the following valid and reliable self-report measures at the beginning and end of the study: Karnofsky Performance Status scale [27], the Self-administered Comorbidity Questionnaire (SCQ) [28], and the AFI [24].

### Objective measures of cognitive function

To evaluate the cognitive function of each survivor, a custom tablet-based cognitive assessment battery was used. The ACE-X measures different aspects of high-order cognitive control abilities (e.g. attention, working memory, speed of information processing, and multi-tasking) [29]. ACE-X has a user-friendly interface, as well as adaptive psychometric staircase algorithms, which modulate the challenge-level of a task on a trial-by-trial basis, based on each individual’s performance until the survivor is performing at ~ 80% rate of accuracy [30–32]. This approach was designed so that comparisons between individuals reflect actual differences in a specific cognitive ability and not disparities in the testing parameters [26]. Critically, this approach removes any biases of age-related slowing, instrumentation, or ceiling/floor effects, and finds an individualized level of performance that is specific to each user. Calculation of these baseline levels leads to the creation of within-task indices (a single number) for each cognitive construct that is presented at the end of each task. In the current study, three modules (i.e., Sustained Attention Ace Task (SAAT): attention; Spatial Span: working memory; Tap and Trace: multitasking) were selected to assess specific cognitive control abilities. Recent work from Dr. Anguera’s laboratory determined normative data for healthy individuals who are 40 + years of age for the three modules used in this study (i.e., SAAT = 445.8 ms (ms) (± 143.27), Spatial Span = 3631.4 ms (± 947.2), Tap and Trace = 919.1 ms (± 575.04); manuscript in review). The total time to complete the three modules was approximately 15 min. Previous work using non-adaptive mobile assessments involving 15,000 participants concluded that mobile assessment methods show comparable performance outcomes relative to traditional lab/clinic-based diagnostics [33], confirming mobile assessments as a robust methodology.

### Interventions

The TCI that was evaluated in this pilot study (i.e., EVO or AKL-T01) is derived from a TCI called NeuroRacer that demonstrated efficacy in enhancing cognitive function in older adults [20]. The intervention involves guiding a character through an immersive environment while responding to select targets, with the design format being ideally entertaining and enjoyable to pediatric [34] and geriatric [20] populations. The TCI utilizes a proprietary set of adaptive game algorithms that makes it function at a level of difficulty that is continually personalized to the user. This approach ensures the user’s engagement and that the TCI is properly titrated for each individual, allowing room for the user to improve over time. Based on the significant findings in three independent samples [20, 35, 36], survivors were instructed to use the TCI for 25 min a day, 5 days a week, for one month. The TCI application was set to automatically conclude each daily session after 25 min.

The EMACI is a word jumble game that was designed to test and train one’s verbal fluency by tracing words with two or more letters in any direction with a grid of letters. The EMACI has an adaptive element to it (i.e., it changes difficulty as participants complete a set number of trials). However, unlike the TCI, this adaptivity does NOT occur on a trial-by-trial basis and it does NOT scale proportionally with performance. While the EMACI was designed to be as equally immersive and fun as the TCI, using a survey of expectancy, 70 adults were asked to rate the efficacy of the two interventions. No differences were found in the expectancy measures for the efficacy of the two interventions (i.e., improvements in attention), as well as for having fun and for feelings of well-being (unpublished data). These findings suggest that any benefits from the TCI, that would be found in the current pilot RCT, could not be attributed solely to placebo effects.

### Study procedures

The study was approved by the Committee on Human Research at the University of California, San Francisco and the Protocol Review Committee of the Helen Diller Family Comprehensive Cancer Center. The study was registered on ClinicalTrials.gov (NTC04870320).

During the 30-min orientation meeting (via Zoom or phone), the survivors were given their login information and briefly oriented to the ACE-X and intervention applications. In addition, they received information on study activities and timelines and were encouraged to ask questions. Survivors were asked to complete the ACE-X immediately after the orientation call and begin their first intervention session before the end of that day. This orientation meeting was Day 1 of the 28-day study.

Survivors were instructed to use their intervention at least five days per week. Both the TCI and EMACI timed out after approximately 25 min of use and reset at midnight for the following day. After each week of the four-week study, survivors were asked to complete surveys about any adverse effects they experienced while using the intervention. Any reports of even mild adverse effects were addressed by the study staff. At two weeks after initiation of the intervention (~ 14 days) and at the end of the study (~ 28 days), survivors received an email to complete the study’s self-report measures and the ACE at that time. After the study, survivors mailed their iPad back to the study office and were compensated with a $50 e-code to a retailer.

### Sample size calculation

The original sample size estimate was calculated for the primary outcomes: attention and working memory. A sample size of 60 (30 in each group) would achieve 80% power to detect a Cohen’s d effect size of 0.35 (between a weak and medium effect), with a 2-sided alpha of 0.05, beta of 0.20, and correlation of 0.30 between the pre- and post-test measures [37]. Effect size calculations were performed using PASS 13 [38].

## Analysis

Descriptive statistics and frequency distributions were generated using IBM SPSS Statistics Version 28 (IBM Corporation, Armonk, NY). Differences in demographic and clinical characteristics between the TCI and EMACI groups were evaluated using parametric and non-parametric tests. To evaluate for between group differences in objective measures of cognitive function (i.e., attention, working memory, multi-tasking) multilevel regression analyses were performed [39, 40]. Specifically, because the outcome measures were nonnormal continuous outcomes, multilevel regression analysis was done using the bootstrap procedure for estimation [41–44]. This approach allowed for the interpretation of the between group differences pre and post the intervention, retaining the scale of the outcome measures.

In addition, these analyses employed the nonparametric bootstrap in order to obtain non-parametric bias-corrected bootstrapped confidence intervals (BC CI) using 2,500 repetitions [41]. When BC CI are used to draw conclusions about statistical significance, the regression coefficient or other effect is considered to be significant if zero is not in the interval. Effect estimates are reported using marginal means estimated from the bootstrapped MLReg model (Stata 17, program *mixed*). The use of MLReg for examining longitudinal change allows unbiased estimation even if participants miss one or more measurement occasions, as long as the missingness is ignorable [45–48]. This approach is possible through the use of Full Information Maximum likelihood [49, 50] and the Expectation-Maximization algorithm [49, 51].

## Results

### Feasibility

**Fig. 1:**
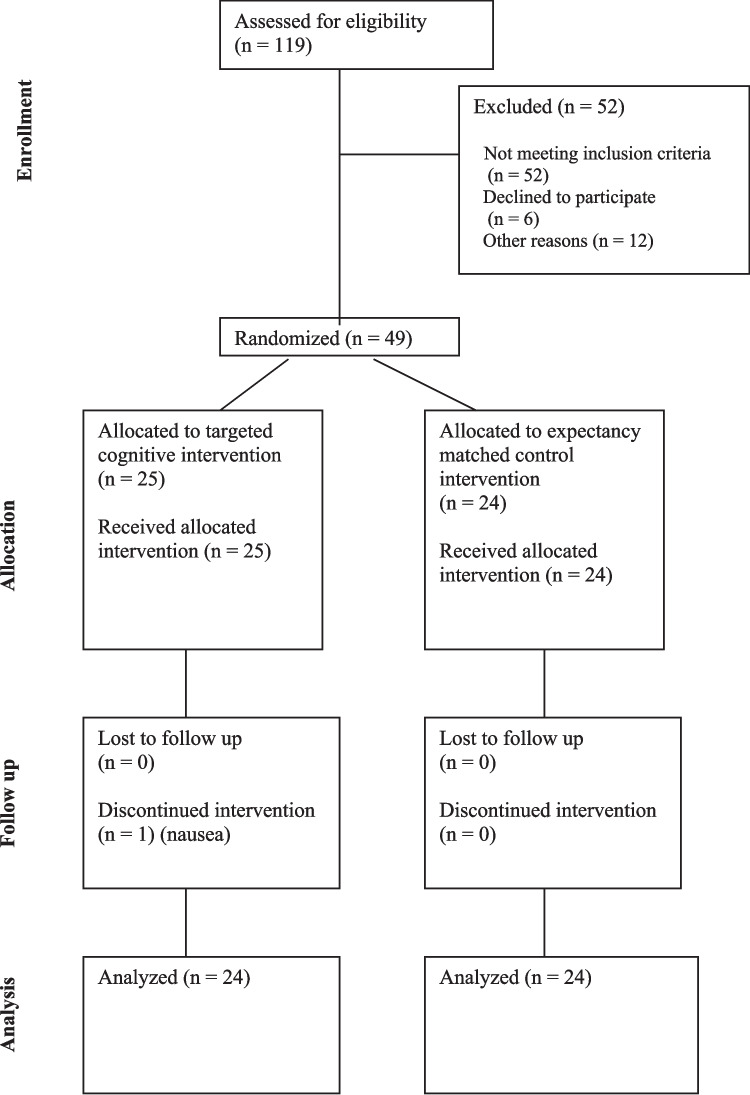
CONSORT flow diagram of the study

As shown in Fig. 1, of the 119 who survivors who were screened, 52 were not eligible (48 with an AFI score of > 7.5, 4 receiving treatment); 6 declined to participate; and 12 were not able to participate for a variety of reasons (e.g., vacation plans). The remaining 49 survivors were randomized to the TCI (*n* = 25) or EMACI (*n* = 24) groups. Of these 49 survivors, one survivor in the TCI group withdrew from the study.

### Sample characteristics

Except for KPS score, no differences were found between the TCI and EMACI groups on any demographic and clinical characteristics (Table 1). Compared to the TCI group, survivors in the EMACI group had a lower KPS score at enrollment.

**Table 1:** Differences in demographic and clinical characteristics between the Targeted Cognitive Intervention (TCI) and Expectancy Matched Active Control Intervention (EMACI) at enrollment

Characteristic	TCI Group 50.0% (*n* = 24)	EMACI Group 50.0% (*n* = 24)	Statistics
	Mean (SD)	Mean (SD)	
Age (years)	55.8 (13.4)	58.2 (11.1)	t = −0.68, *p* = .497
Time since diagnosis (years)	9.4 (5.3)	8.6 (4.8)	t = 0.51, *p* = .613
Karnofsky Performance Status score	93.3 (9.2)	84.2 (11.4)	t = 3.07, *p* = .004
Number of comorbidities	1.2 (1.0)	1.5 (1.2)	t = −0.77, *p* = .444
Self-administered comorbidity score	2.1 (1.7)	2.8 (2.6)	t = −1.06, *p* = .295
Attentional Function Index score	6.1 (0.9)	5.7 (0.8)	t = 1.52, *p* = .136
	% (n)	% (n)	
Female (% yes)	91.7 (22)	100.0 (24)	FE, *p* = .489
Education			
Some college	16.7 (4)	16.7 (4)	Χ^2^ = 3.18, *p* = .364
College graduate	29.2 (7)	20.8 (5)	
Some graduate school	4.2 (1)	20.8 (5)	
Advanced degree	50.0 (12)	41.7 (10)	
Married/partnered (% yes)	66.7 (16)	60.9 (14)	FE, *p* = .766
Lives alone (% yes)	20.8 (5)	26.1 (6)	FE, *p* = .740
Race/ethnicity			
Asian	16.7 (4)	4.2 (1)	U, *p* = .913
White	66.7 (16)	79.2 (19)	
Mixed ethnic background	12.5 (3)	12.5 (3)	
Hispanic	4.2 (1)	4.2 (1)	
Annual household income			
$20,00 to $39,999	4.5 (1)	5.0 (1)	U, *p* = .396
$40,000 to $59,999	4.5 (1)	5.0 (1)	
$60,000 to $79,999	27.3 (6)	15.0 (3)	
$80,000 to $99,000	22.7 (5)	20.0 (4)	
$100,000 or more	40.9 (9)	55.0 (11)	
Currently employed (% yes)	62.5 (15)	58.3 (14)	FE, *p* = 1.000
Exercise on a regular basis (% yes)	91.7 (22)	83.3 (20)	FE, *p* = .666
Past or current smoker (% yes)	25.0 (6)	33.3 (8)	FE, *p* = .752
Cancer diagnosis			
Breast	87.5 (21)	87.5 (21)	Χ^2^ = 6.00, *p* = .199
Colon	0.0 (0)	4.2 (1)	
Non-Hodgkin’s lymphoma	4.2 (1)	0.0 (0)	
Ovarian	0.0 (0)	8.3 (2)	
Other	8.3 (2)	0.0 (0)	
Metastatic disease (% yes)	26.1 (6)	30.4 (7)	FE, *p* = 1.000
Comorbid conditions (% yes)			
Heart disease	0.0 (0)	4.2 (1)	n/a
High blood pressure	16.7 (4)	25.0 (6)	FE, *p* = .724
Lung disease	8.3 (2)	0.0 (0)	FE, *p* = .489
Diabetes	4.2 (1)	0.0 (0)	n/a
Ulcer or stomach disease	4.2 (1)	1.3 (1)	FE, *p* = 1.000
Kidney disease	0.0 (0)	0.0 (0)	n/a
Liver disease	0.0 (0)	0.0 (0)	n/a
Anemia or blood disease	4.2 (1)	0.0 (0)	n/a
Depression	16.7 (4)	20.8 (5)	FE, *p* = 1.000
Osteoarthritis	12.5 (3)	8.3 (2)	FE, *p* = 1.000
Back pain	17.4 (4)	37.5 (9)	FE, *p* = .193
Rheumatoid arthritis	4.2 (1)	4.2 (1)	FE, *p* = 1.000

Abbreviations: FE = Fischer’s Exact, n/a = not applicable, SD = standard deviation, U = Mann Whitney U test

### Use of video games and expectancy measures at enrollment

No differences were found between the two groups in the previous or current use of video or puzzle or word games (Table 2). In addition, no differences were found between the two groups in any of the expectancy questions (Table 2).

**Table 2:** Differences in use of video games and expectancy questions between the Targeted Cognitive Intervention (TCI) and Expectancy Matched Active Control Intervention (EMACI) at enrollment

Questions	TCI Group 50.0% (*n* = 24)	EMACI Group 50.0% (*n* = 24)	Statistics
	% (n)	% (n)	
Use of Video Games Questions			
Not including the past year, were you at one point a regular video game player?			
Yes – regular video games	12.5 (3)	4.8 (1)	Χ^2^ = 0.85, *p* = .838
Yes – puzzle and word games	29.2 (7)	33.3 (7)	
Yes – both video and puzzle and word games	8.3 (2)	9.5 (2)	
No	50.0 (12)	52.4 (11)	
Do you currently play video games?			
Yes – regular video games	4.2 (1)	4.5 (1)	Χ^2^ = 0.85, *p* = .838
Yes – puzzle and word games	37.5 (9)	27.3 (6)	
Yes – both video and puzzle and word games	4.2 (1)	9.1 (2)	
No	54.2 (13)	59.1 (13)	
Expectancy Questions			
	Mean (SD)	Mean (SD)	
Confidence that the assigned game will result in improved performance on the ACE test four weeks from now	6.2 (2.9)	6.0 (2.0)	t = 0.23, *p* = .820
Belief that the assigned game will help you improve your general cognitive function	5.8 (3.0)	5.9 (1.8)	t = −0.11, *p* = .917
Faith that stress management training will improve your cognitive function	5.9 (2.7)	6.1 (2.4)	t = −0.29, *p* = .774
Faith that self-improvement programs will improve your cognitive function	5.6 (2.7)	6.1 (1.9)	t = −0.63, *p* = .535
Faith that medical care will improve your cognitive function	4.2 (2.6)	4.7 (2.0)	t = −082, *p* = .418

Confidence rating – 0 = I have no confidence to 10 = I have complete confidence

Belief rating – 0 = I don’t believe it to 10 = I 100% believe it

Faith rating – 0 = No faith to 10 = total faith

Abbreviations: SD = standard deviation

### Effects of the TCI on attention, working memory, and multi-tasking

**Fig. 2:**
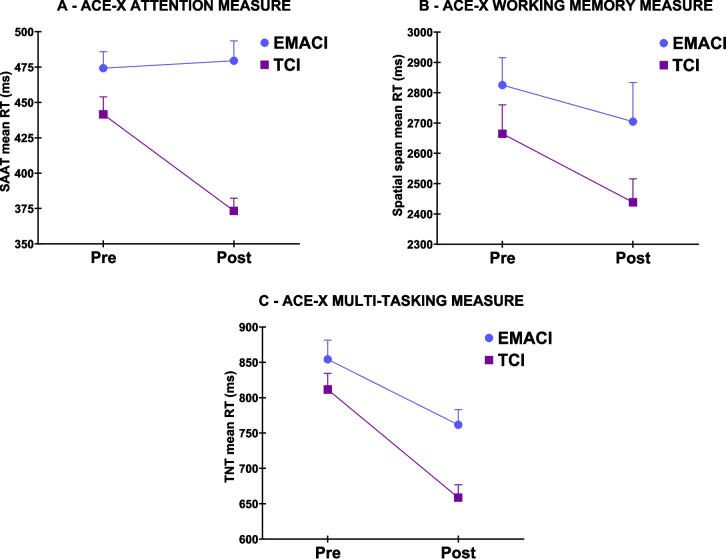
Changes over time in pre and post test scores for the Adaptive Cognitive Evaluation Explorer (ACE) measures of attention (Sustained Attention Ace Task) (SAAT), (**A**), working memory (i.e., spatial span, (**B**)), and multi-tasking (i.e., TNT, (**C**)) between the targeted cognitive intervention (TCI) and expectancy matched active control intervention (EMACI). Values are plotted as means and foot strapped standard errors. Abbreviations: ms = milliseconds, RT = reaction time

For all three cognitive tests, no differences were found between the TCI and EMACI groups at enrollment. As shown in Fig. 2A, a significant omnibus test for the cross-level (group by time) interaction was found for the ACE-X sustained attention measure (i.e., SAAT mean reaction time (RT)) (Χ^2^ = 10.36, 2df, *p* < 0.01). An examination of the simple interaction effects revealed that the groups differed for the contrast between the pre and post assessments (contrast = −73.5, BC CI = −119.5, −28.8). An examination of the simple main pairwise effects for time within the TCI group revealed that the means decreased significantly from the pre to the post assessment (contrast = −68.27, BC CI = −112.27, −24.28). An examination of the simple main effects for the pairwise differences across time within the EMACI group revealed no significant differences.

As shown in Fig. 2B, for the ACE-X working memory measure (i.e., spatial span mean RT), no significant simple effects were found for either the TCI or EMACI groups. While no cross-level interactions were found, as shown in Fig. 2C, for the ACE-X multi-tasking measure (i.e., triangle trace, TNT), significant simple effects were observed for the TCI group (contrast pre to post = −152.88, BC CI = −232.49, −73.26) and for the EMACI group (contrast pre to post = −92.66, BC CI = −177.28, −8.04).

### Adherence and satisfaction with the TCI and EMACI

No between group differences were found in survivors’ level of adherence with the intervention (i.e., TCI = 95.8%, EMACI = 96.0%, *p* = 0.88). In terms of satisfaction with the interventions (Table 3), no differences were found between the two groups in ratings of how challenging the interventions were; ability of the game to improve attention and memory; and motivation to use the intervention. Compared to the TCI group, the EMACI group rated the intervention more enjoyable and less burdensome.

**Table 3:** Differences in satisfaction questions between the Targeted Cognitive Intervention (TCI) and Expectancy Matched Active Control Intervention (EMACI) at end of study

Questions	TCI Group 50.0% (*n* = 24)	EMACI Group 50.0% (*n* = 24)	Statistics
	Mean (SD)	Mean (SD)	
How much did you enjoy playing the study game? (0 = did not enjoy to 10 = enjoyed it greatly)	6.0 (3.2)	9.1 (1.9)	U, *p* < .001
How challenging was the study game? (0 = easy to 10 = difficult)	6.2 (2.2)	5.8 (2.9)	U, *p* = .925
How much do you think playing the game improved your ability to pay attention?^a^	1.4 (1.1)	2.0 (0.8)	U, *p* = .069
How much do you think playing the game improved your ability to remember things?^a^	1.2 (1.0)	1.5 (0.8)	U, *p* = .240
How many days on average did you play your game during the study? (0 to 7 days)	5.9 (0.9)	6.0 (1.4)	U, *p* = .257
How much of a burden was it to be in the study? (0 = no burden whatsoever to 10 = extremely high burden)	3.1 (2.6)	1.2 (1.8)	U, *p* = .011
How would you rate your motivation to play your study game at the beginning of the study? (0 = no motivation to 10 = extremely high motivation)	8.1 (1.7)	8.5 (2.0)	U, *p* = .301
How much of a burden was it to complete the ACE assessment (0 = no burden whatsoever to 10 = extremely high burden)	2.5 (2.7)	3.3 (2.6)	U, *p* = .180
How much of a burden was it to complete the weekly surveys? (0 = no burden whatsoever to 10 = extremely high burden)	1.5 (1.9)	1.2 (1.8)	U, *p* = .489
How much of a burden was it to play your game 25 min per day, 5 days per week? (0 = no burden whatsoever to 10 = extremely high burden)	3.5 (2.8)	1.5 (2.4)	U, *p* = .004

^a^0 = not at all, 1 = a little bit, 2 = somewhat, 3 = a lot, 4 = a whole lot

Abbreviations: SD = standard deviation, U = Mann Whitney U test

### Treatment-related adverse effects

Over the 4 weeks of the study, eye strain was reported by zero and four survivors in the EMACI and TCI groups, respectively. Two of the survivors in the TCI group reported nausea and one of them withdrew from the study. In terms of muscle or joint aches, five survivors in the TCI and one in the EMACI reported this adverse effect. Except for the survivor who withdrew, for all of the adverse effects, the severity ratings were mild and none of them required an intervention.

## Discussion

Findings from this pilot RCT of a TCI compared to an EMACI in cancer survivors suggest that the intervention was feasible and acceptable. Of note for the ACE-X measure of attention, a significant group x time interaction was found in favor of the TCI group. In addition, while improvements in muti-tasking occurred in both groups, while not statistically significant, the trend was larger in the TCI group (i.e., −152.88 versus −92.66). Equally important, in both groups, adherence with the intervention was high and adverse effects were minimal. These preliminary findings provide promising evidence of feasibility, acceptability, and efficacy that warrant evaluation in a RCT with a larger sample of cancer survivors.

While no between group differences were found in the working memory measure, improvements in attention and multitasking are consistent with previous findings using the TCI in older adults [20, 52, 53]. These preliminary findings are encouraging given that the most common self-reported problems in survivors with CRCI include difficulties with attention and multi-tasking [54–59]. One of the advantages of our TCI is that it incorporates quantitative measurements that reflect a person’s current state as the input arm of the loop (i.e., performance) to guide real time adjustments of stimuli, rewards, and difficulty of the intervention to close the loop. This closed loop TCI is optimized to maintain a person’s engagement with the intervention. As the person’s performance improves over time, the TCI becomes more difficult. However, if the challenge is pushed too far and a high level of performance is not sustained, the TCI becomes easier. The closed loop system maintains the TCI at a “sweet spot” that maximizes fun and engagement. This approach ensures that the TCI is never so hard that it is frustrating or so easy that it is boring. This approach achieves a maximal amount of engagement for driving neuroplasticity [60].

This pilot RCT was the first to use an EMCI which is recommended for scientifically rigorous studies that use a TCI [61–63]. While no comparative data are available, survivors in both groups rated their beliefs that the assigned game would improve their “general cognitive function” at the completion of the study at approximately 6 on a 0 (I don’t believe it) to 10 (100% believe it) scale. Equally interesting, over 50% of our sample did not play video games on a regular basis before the study. This finding is not surprising given that while the average videogame player is 33 years old, the highest percentage of users are under 18 (24%) and between 18 and 34 (36%) years of age [64]. However, when survivors with CRCI were invited to participate, they were willing to learn more about the study and to be taught how to use the ACE and the gaming technology.

Two equally important study purposes were to evaluate satisfaction with the intervention and adverse effects. As shown in Table 3, compared to survivors assigned to the TCI, those assigned to the EMACI enjoyed it more and found it slightly less burdensome. However, for both groups the ratings of burdensomeness were relatively low and adherence with the interventions was extremely high. When asked to rate their enjoyment with playing their assigned game using a 0 (did not enjoy) to 10 (enjoyed it greatly) scale, 41.6% of the survivors in the TCI group rated it an 8 or above. It is not entirely clear why the survivors in the TCI arm found the game slightly more burdensome. It may be that the TCI required more focused attention and engagement. While the TCI appears to be a typical entertainment-designed video game, it underlying mechanics create a challenging experience that requires dedication to realize beneficial effects. In the larger RCT, more detailed information will be obtained on this component of satisfaction.

Given that no data were available on potential adverse effects from the interventions in a sample of cancer survivors, eye strain, nausea, and muscle and joint aches were evaluated on a weekly basis. Any survivor who reported any adverse effect was contacted by a member of the study staff. The most common adverse effect was mild muscle and joint aches in the TCI group. Staff advised survivors to periodically relax their shoulders during play or alter their position when playing the game. These interventions alleviated these symptoms in 100% of the cases.

As with any pilot study, several limitations warrant consideration. The sample was relatively homogenous in terms of gender and cancer diagnosis. One of the goals of the larger RCT will be to increase the number of male participants and survivors with diagnoses other than breast cancer. Second, the relatively small sample size allowed for only an examination of trends and covariates (e.g., KPS scores) were not included in the analyses. However, the significant cross level interaction for the attention measure does support the need for a full-scale RCT. Finally, given that this TCI demonstrated efficacy in younger and older adults with depression [52] and depression co-occurs in survivors with CRCI [65], the effect of the TCI on common symptoms in cancer survivors warrants evaluation.

Despite these limitations, the findings from this pilot RCT of a TCI compared to an EMACI in cancer survivors suggest that the intervention was feasible, acceptable, and without adverse effects. In addition, while larger studies are needed, the scores for the three measures were within the normative ranges. However, larger samples will be needed to determine clinically meaningful reductions in each of the measures. The preliminary positive results for attention and multi-tasking suggest that this intervention can improve a devastating problem in cancer survivors.

## Data Availability

Data will be made available from the corresponding author after completion of a data sharing agreement with the University of California, San Francisco.
